# Supplementary material for: Prevalence and Epidemiological and Clinical Features of Bacterial Infections in a Large Cohort of Patients Hospitalized for COVID-19 in Southern Italy: A Multicenter Study
Source: Antibiotics (Basel). 2023 Jun 29;12(7):1124. doi: 10.3390/antibiotics12071124 (PMC10376680; doi:10.3390/antibiotics12071124)
Supplement: Supplementary file 1 [file antibiotics-12-01124-s001.zip › antibiotics-2459820-supplementary.pdf]

Supplementary Table S1. Demographic, clinical, and biochemical characteristics of patients who received antimicrobial prescriptions, according to the presence of bacterial infection.

	Bacterial infection	No bacterial infection	P value
**N° of patients**	155	323	
**Mean age (SD), years**	63.2 (16.9)	66.2 (15.5)	0.06
**Males, n° (%)**	106 (68.4)	200 (61.9)	0.17
**Mean length of hospital stay (SD), days**	17.6 (10.6)	16.1 (10.4)	0.12
**Admitted to ID wards, n° (%)**	151 (97.4)	277 (85.8)	**<0.001**
**Charlson Comorbidity Index (median, range)**	3 (4)	3 (4)	0.46
**Comorbidities, n° (%)**			
- **Cardiovascular disease**	49 (31.6)	99 (30.6)	0.88
- **Diabetes**	40 (25.8)	77 (23.8)	0.68
- **COPD**	14 (9)	36 (11.1)	0.46
- **Chronic kidney disease**	25 (16.1)	34 (10.5)	0.08
- **Chronic liver disease**	12 (7.7)	12 (3.7)	0.09
- **Malignancies**	14 (9)	29 (9)	0.99
- **HIV**	2 (1.3)	1 (0.3)	0.21
**Severity of COVID-19 disease, n° (%)**			
- **Mild or Moderate**	67 (43.2)	100 (31)	**0.008**
- **Severe/Critical**	88 (56.8)	223 (69)	
**SOFA score (median, IQR)**	1 (4)	3 (2)	**<0.001**
**Supplemental oxygen therapy, n° (%)**			
- **None**	20 (12.9)	24 (7.4)	**<0.001**
- **Nasal cannula or simple face mask**	95 (61.3)	125 (38.7)	
- **HFNC**	17 (11)	32 (9.9)	
- **CPAP/NIV**	19 (12.3)	133 (41.2)	
- **Mechanical ventilation**	3 (1.9)	6 (1.9)	
**Baseline laboratory parameter (mean, SD)**			
- **WBC (x1000/µl)**	10.08 (9.4)	9.8 (4.2)	0.74
- **Lymphocytes count (x1000/µl)**	1.6 (6.9)	1.0 (0.6)	0.23
- **INR**	1.2 (0.5)	1.2 (0.8)	0.33
- **Creatinine (mg/dl)**	1.6 (1.8)	1.2 (1.2)	0.32
- **ALT (UI/ml)**	56.8 (81.3)	52.4 (85.4)	0.60
- **Total bilirubin (mg/dl)**	0.8 (1.2)	0.9 (2.2)	0.16
- **PCR (x ULN)**	15.6 (49)	23.3 (105)	0.38
**Corticosteroid treatment, n° (%)**	142 (91.6)	303 (93.8)	0.51
